# Radiation-induced sarcomas of the head and neck in post-radiation nasopharyngeal carcinoma

**DOI:** 10.1007/s11547-016-0695-5

**Published:** 2016-10-13

**Authors:** Qiuxia Yang, Yunxian Mo, Qianqian Zhao, Xiaohua Ban, Mingyan He, Peiqiang Cai, Xuewen Liu, Chuanmiao Xie, Rong Zhang

**Affiliations:** 1 Department of Medical Imaging Center, State Key Laboratory of Oncology in South China, Collaborative Innovation Center for Cancer Medicine, Sun Yat-sen University Cancer Center, 651 Dongfeng Road East, Guangzhou, 510060 China; 2 Department of Radiology, The First Affiliated Hospital, Sun Yat-sen University, Guangzhou, 510086 China

**Keywords:** Nasopharyngeal carcinoma, Radiation-induced sarcoma, CT, MRI, Prognosis

## Introduction

Radiotherapy (RT) is the curative treatment modality for nasopharyngeal carcinoma (NPC), which is a common tumor in the southern Chinese population. However, ionizing radiation is itself a known carcinogen. There is a large population at risk of developing sarcomas in the irradiated fields. Compared with post-radiation squamous cell carcinoma (SCC) [1], radiation-induced sarcomas (RIS) of the head and neck following RT for NPC are even less common, with a crude incidence in adults of less than 0.5 % in various situations [1–5]. With improved patient survival [6–8], RIS is likely to be encountered more frequently. It is now generally believed that complete surgical resection of RIS provides the only chance of a cure [3, 4]. Unfortunately, the reported prognosis is poor owing to advanced RIS at diagnosis [3, 4, 9]. RIS has become a critical problem that can limit long-term survival and hinder quality of life. To detect an RIS at an earlier stage, the radiologist should be aware of the common radiological appearances, which can be scrutinized on follow-up scans.

The purpose of this study was to document the clinical findings and the radiological features on computed tomography (CT) and magnetic resonance imaging (MRI) of RIS in the head and neck following RT for NPC.

## Materials and methods

### Patients

**Fig. 1:**
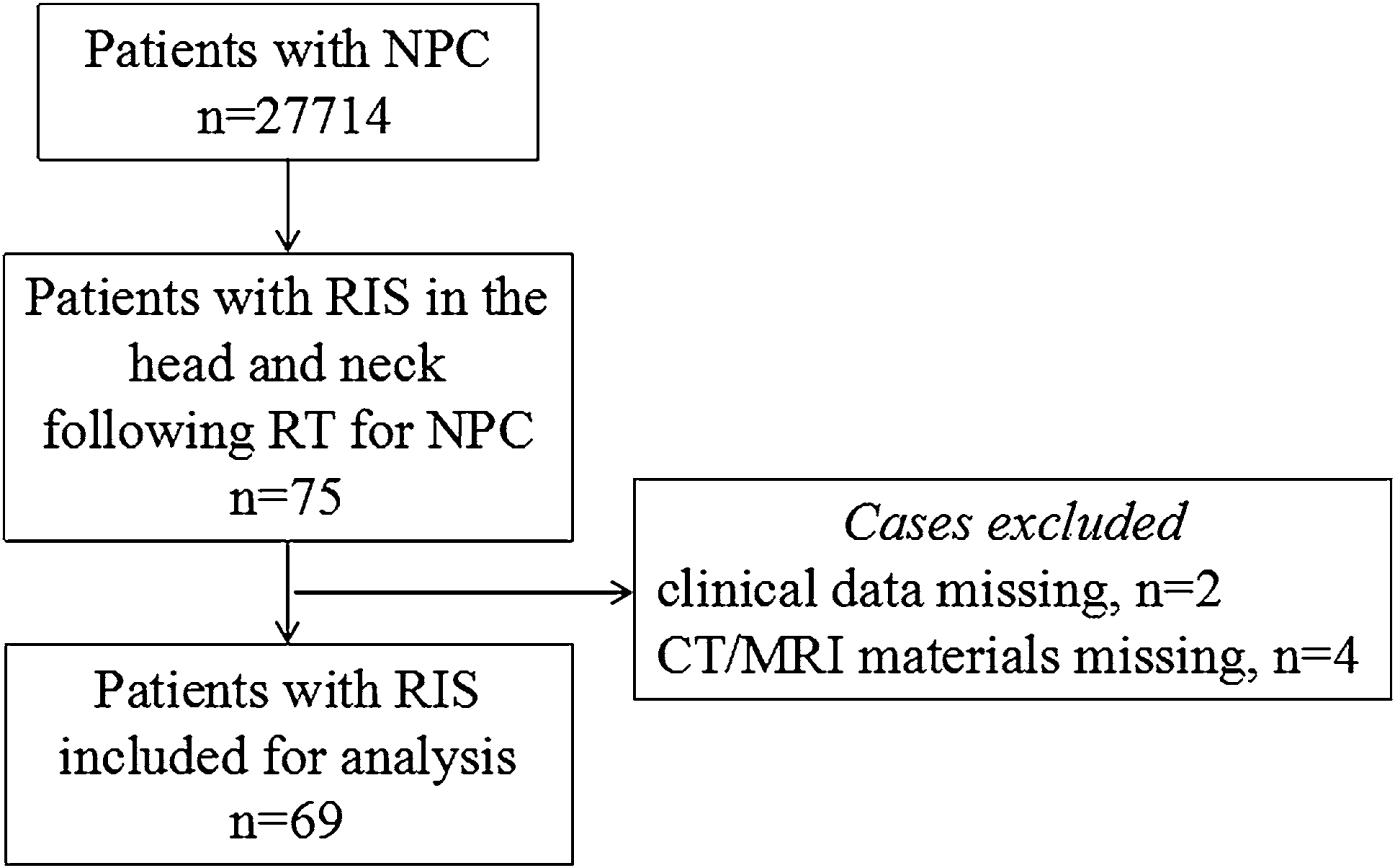
Flowchart showing patient recruitment

The institutional ethics review board approved this retrospective study and waived the need for informed consent. Patients with RIS in the head and neck following RT for NPC identified from a review of the histological and radiological results from December 1998 to September 2012 met the inclusion criteria. The clinical records were reviewed to determine histology, latency period, clinical presentation and treatment. The diagnostic criteria of patients with RIS was based on the criteria published by Cahan et al. [10]: a prior history of RT; the occurrence of a sarcoma within the previously irradiated field; a latency between irradiation and the second primary sarcoma of at least 3 years; histologically proven sarcoma arising within the irradiated field. One of the co-authors engaged in the pathological diagnosis. Patients of RIS without CT or MRI materials in our institution or without clinical data were excluded (Fig. 1).

### Radiation techniques

The radiation techniques that were used in this study had been described in detail by other colleagues in our hospital [11, 12]. The radiation fields for the nasopharynx normally included two opposite preauricular fields. For patients with nasal cavity or poststyloid area invasion, an anterior or postauricular supplementary field was added. At the cervical region, the anterior and posterior split fields were used in the first period and perpendicular small opposing fields in the second. In this study, the primary nasopharyngeal tumors were treated with a dose of 40–90 Gy with the average dose of 68.7 Gy, and the neck received 37–70 Gy with the average dose of 57.7 Gy, depending on the lymph nodes involvement.

### CT and MRI examinations

A 16-slice spiral CT (Brilliance TM 16, Philips Medical Systems, the Netherlands) or a dual-slice CT (Twin FLASH, Philips Medical Systems, the Netherlands) was used for multiphasic CT scans (i.e., nonenhanced phase, arterial and venous phases). Other parameters were as follows: section thickness, 5 or 10 mm; gap, 5 or 10 mm; tube current, 250 mA; and tubular voltage, 125 kV. The contrast-enhanced CT scanning was performed after an intravenous injection of iopromide at a dosage of 1.5 ml/kg of body weight (Ultravist 300, Schering, Germany).

The MRI was performed using a 1.5 T superconductive unit (Signa Horizon LX Highspeed, General Electric Medical Systems, USA) with a head coil and a neck coil or a combined head and neck coil. The sequences included spin echo sagittal, coronal and transverse T1-weighted images (TR/TE = 2000 ms/120 ms) and fast spin echo sagittal T2-weighted images (TR/TE = 450 ms/15 ms). Contrast-enhanced sagittal, coronal and transverse T1-weighted images were obtained after the intravenous injection of gadopentetate dimeglumine (Magnevist, Schering, Berlin, Germany) at a dosage of 0.2 mmol/kg of body weight. The fat-suppression technique was used for the gadolinium-enhanced T1-weighted coronal images in 15 patients.

Among the 69 patients, 41 cases underwent CT examination only, 21 cases MRI only, and the remaining 7 cases both CT and MRI scans. In all patients, contrast-enhanced examinations were performed.

### Images evaluation

CT and MRI images were reviewed by the two experienced head and neck authors (one with more than 20 years and another with 4 years of experience, respectively) and decisions were reached by consensus.

Images were assessed with an emphasis on the following aspects: (a) the site or origin of lesions; (b) lesion size (measured at its greatest diameter); (c) density/signal intensity of lesions and pattern, degree of enhancement; (d) extent of lesions including bone destruction.

## Results

### Patients

A total of 69 patients (from 27714 patients) with histological evidence of RIS were enrolled, including 46 men and 23 women with a ratio of 2:1. The median age at the diagnosis of RIS was 48 years (year) (range 21–71 years). Patients’ characteristics at diagnosis of NPC are shown in Table 1.

**Table 1:** Patient characteristics at diagnosis of nasopharyngeal carcinoma (*n* = 69)

Characteristic	No. of patients	%
Sex		
Men	46	66.67
Women	23	33.33
Age (year)		
≤30	15	21.74
>30	54	78.26
TNM stage^a^		
I/II	24	34.78
III/IV	32	46.38
Histological type^b^		
WHO type I	0	
WHO type II and III	57	82.61
Radiation technique^b^		
Conventional radiotherapy	57	82.61
IMRT	0	
Radiation dose (Gy)^b^		
≥55	56	81.16
<55	1	1.45

*WHO* World Health Organization, *IMRT* intensity-modulated radiotherapy

^a^13 cases and ^b^12 cases without records, respectively

### Clinical presentation and latency

The symptoms were related to the sites of RIS. Among them, 42 patients presented with well-marked mass in the maxillofacial region and neck accompanied by pain of certain degrees, 7 patients with serious loss of vision and 20 patients with nasal obstruction and/or bleeding similar to the symptoms of primary NPC.

The average latency period from the end of radiation therapy to the diagnosis of RIS was 10.8 years (range 3–37 years). Most of RIS (44.9 %) occurred at 5–10 years after RT, and up to 87.0 % of RIS developed within 15 years after RT (Table 2). The median latency of RIS originating in the maxillofacial region and in the neck was similar, at 10.8 years with a range of 3–37 and 5–19 years, respectively.

**Table 2:** The latency period from the end of radiation therapy to the diagnosis of radiation-induced sarcomas (*n* = 69)

Characteristic	No. of patients	%
Latency (year)		
3–5	12	17.39
5–10	31	44.93
10–15	17	24.64
15–20	4	5.79
>20	5	7.25

### Imaging features

The main characteristic of the 69 RIS on CT and MRI was soft tissue mass formation. All cases showed heterogeneous density/signal intensity before and after intravenous administration of contrast medium, and 43 of them had marked enhancement compared with adjacent muscles. Most of the solid areas of RIS showed high T2 signal intensity on MRI. 82.6 % of the lesions (*n* = 57) were ill defined, widely extending to adjacent spaces and had bone destruction or blood vessel infiltration. The lesion sizes ranged from 0.7 to 8.2 cm with the average diameter of 4.3 cm, and lesions ≥3 cm accounted for 63.8 %, lesions ≥5 cm accounted for 30.4 %.

**Fig. 2:**
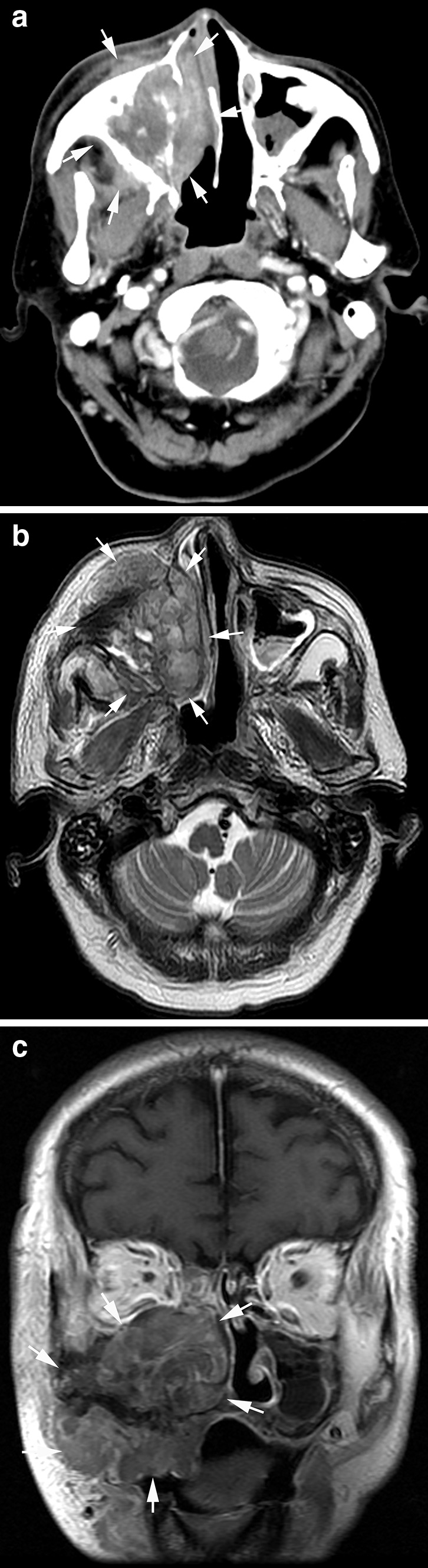
Radiation-induced osteosarcoma 17 years after radiotherapy of a 58-year-old man. **a** Axial contrast-enhanced CT image showed a large heterogeneous tumor in the right maxillary sinus invading the nasal cavity (*arrows*); **b** axial T2-weighted image showed heterogeneously high T2 signal intensity of the tumor; **c** coronal T1-weighted contrast-enhanced image showed the mildly enhanced tumor with wide invasion into the right maxillary region (*arrows*)

**Fig. 3:**
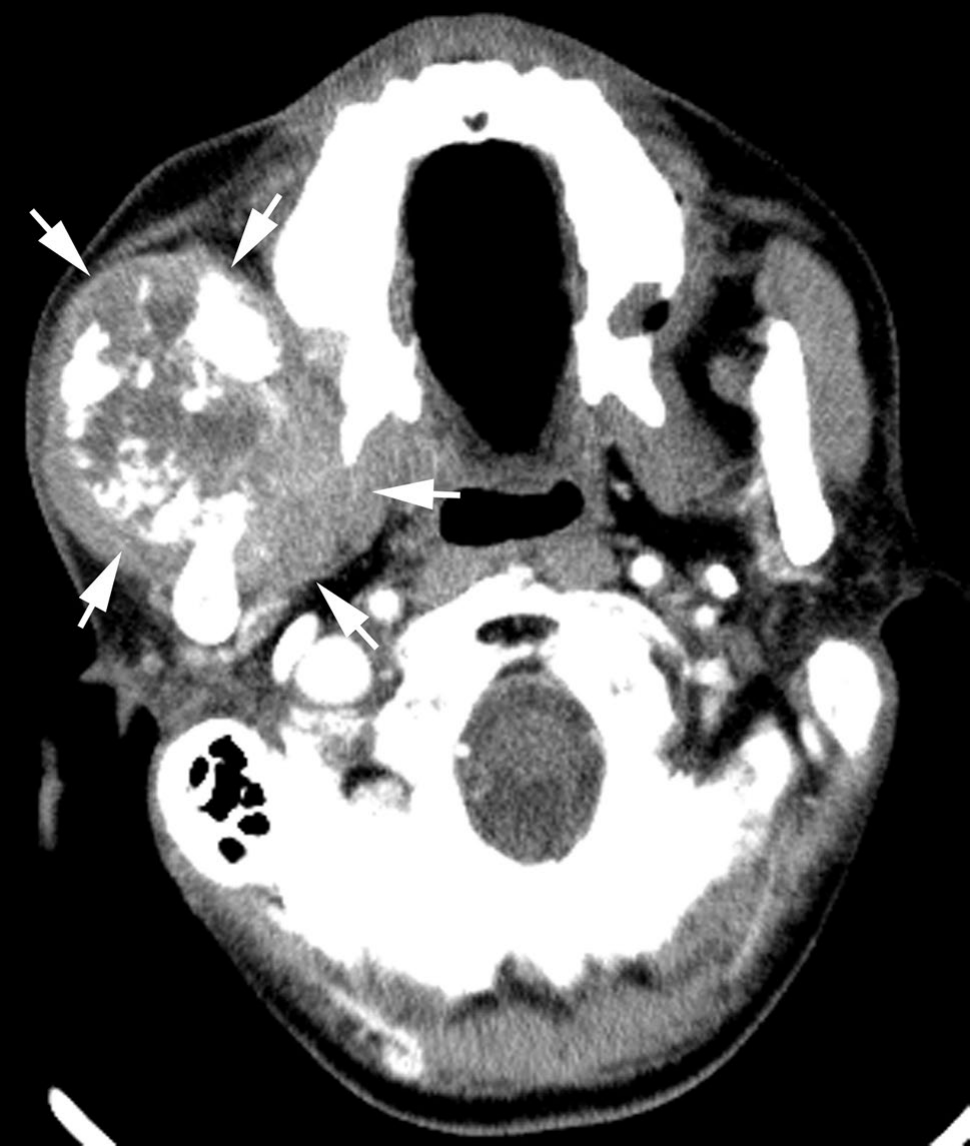
Radiation-induced osteosarcoma 10 years after radiotherapy of a 53-year-old man. Axial contrast-enhanced CT image showed a large heterogeneous tumor, with a large amount of tumor bone formation, arising from the right mandibular ramus destroying the right masseter muscle and medial pterygoid muscle (*arrows*)

**Fig. 4:**
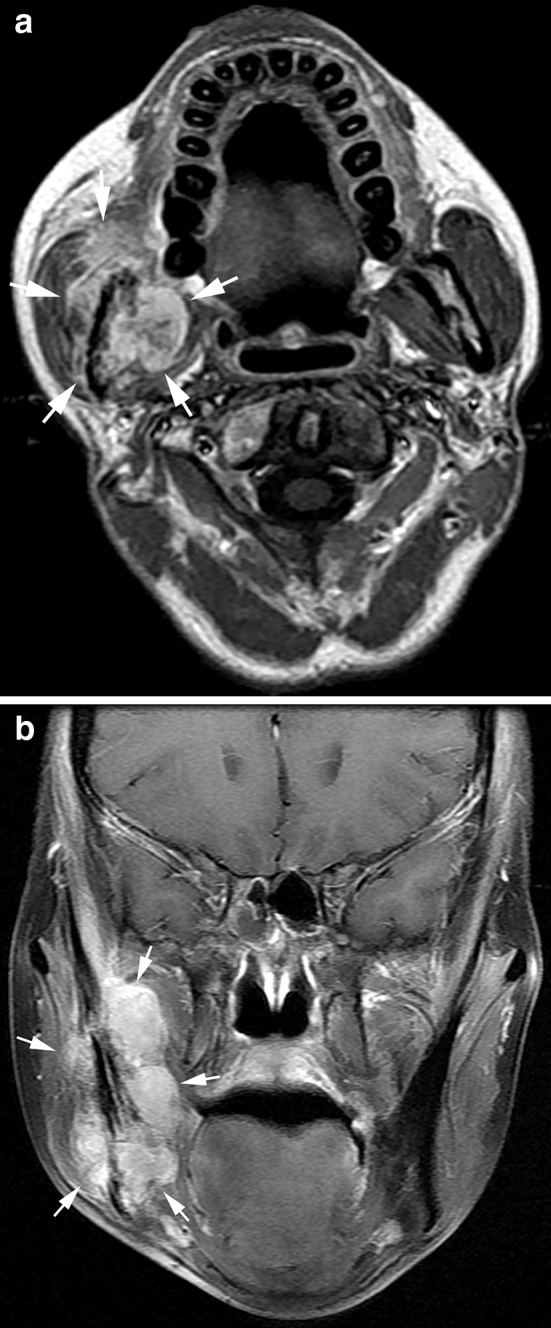
Radiation-induced fibrosarcoma 5 years after radiotherapy of a 35-year-old woman. Axial T1-weighted contrast-enhanced image (**a**) and coronal fat-suppressed T1-weighted contrast-enhanced image (**b**) showed a large heterogeneously marked enhanced tumor of the right mandibular ramus with bone destruction and wide invasion into the muscles (*arrows*)

**Fig. 5:**
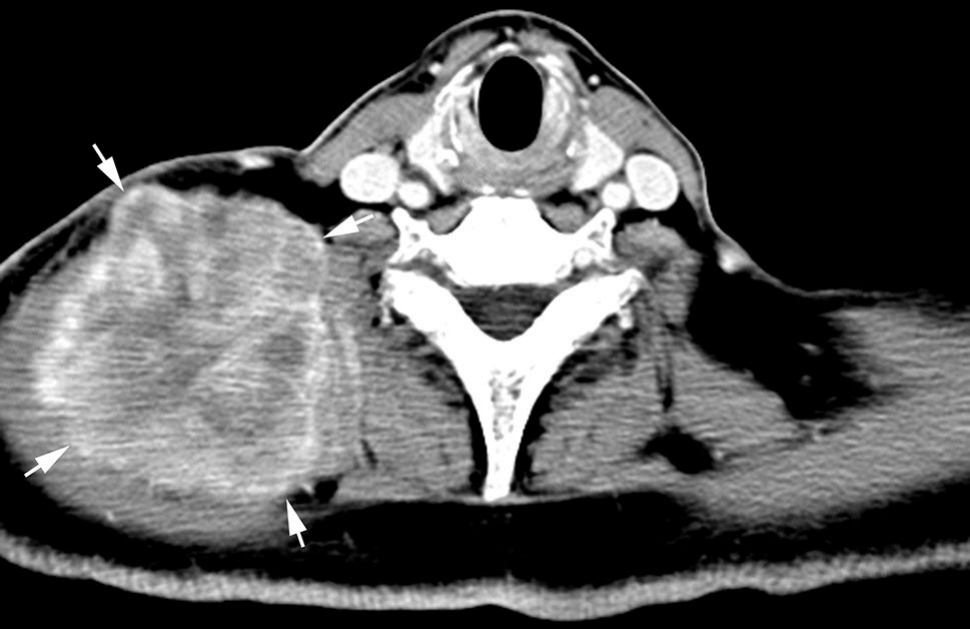
Radiation-induced fibrosarcoma 10 years after radiotherapy of a 50-year-old man. Axial contrast-enhanced CT image showed a large heterogeneously marked enhanced tumor with wide invasion into the muscles in the right lower neck (*arrows*)

**Fig. 6:**
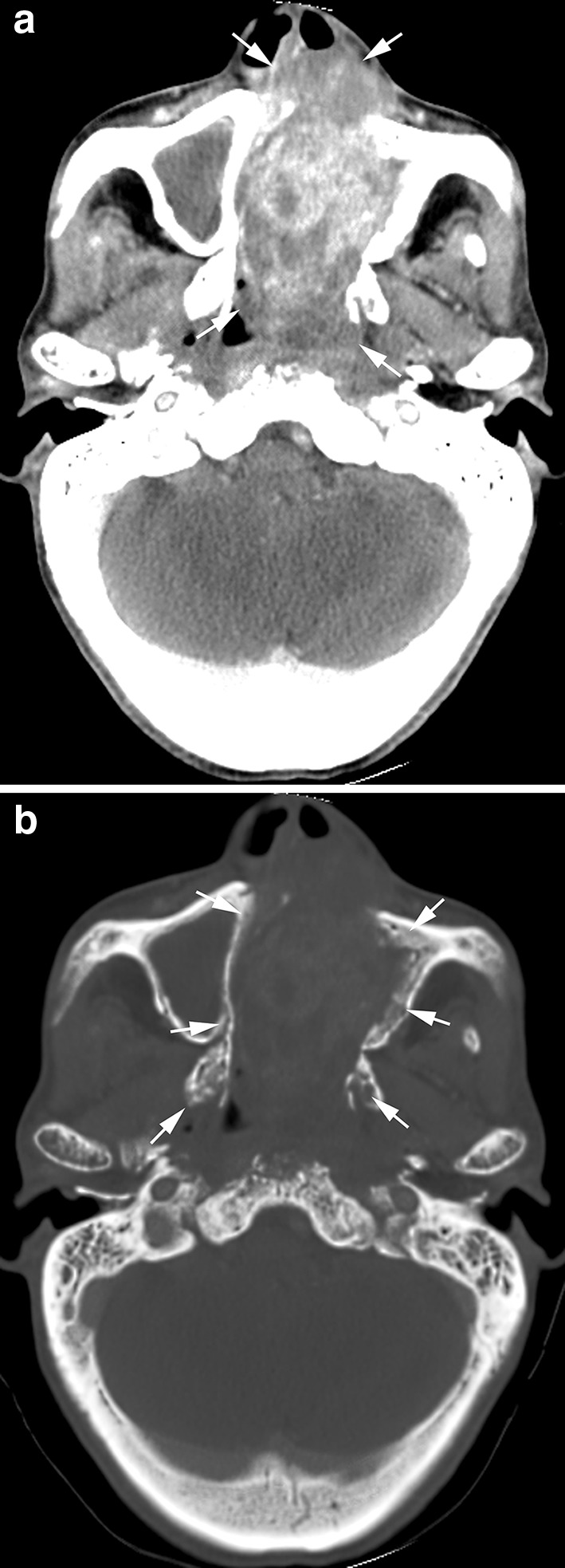
Radiation-induced undifferentiated pleomorphic sarcoma 13 years after radiotherapy of a 40-year-old man. Axial contrast-enhanced CT image (**a**) showed a large heterogeneously marked enhanced tumor in the nasal cavity and left maxillary sinus (*arrows*); axial CT on bony window (**b**) of bone destruction

RIS in the current study showed variations in location and histological subtype. The locations and histologic subtypes of RIS are presented in Table 3 (Figs. 2, 3, 4, 5, 6). All the sarcomas in this study occurred at sites, while in the irradiated field, away from the margins of the original tumor of NPC and the metastatic lymph nodes. The most common origin sites of RIS were the maxillary sinus (*n* = 20), neck (*n* = 14) and mandibular ramus (*n* = 11). The largest histological group was osteosarcoma (*n* = 24), followed by fibrosarcoma (*n* = 22) and undifferentiated pleomorphic sarcoma (*n* = 8). The clinically determined site of RIS was superficial (cutaneous or subcutaneous) in 39 patients and deep in 30 patients. RIS originated in bone in 48 patients with the common histologic types of osteosarcoma (*n* = 24) and fibrosarcoma (*n* = 10) and in soft tissue in 21 patients with the common subtype of fibrosarcoma (*n* = 12), respectively. Among the 24 osteosarcomas, a large amount of tumor bone formation was seen in 10 cases. A subgroup comparison of radiological findings between osteosarcoma and fibrosarcoma is summarized in Table 4.

**Table 3:** Locations, histologic types and imaging findings of radiation-induced sarcomas (*n* = 69)

Histology	No.	%	Median age^a^ (year)	Latency (year)	Site^b^
Osteosarcoma	24	34.78	49	10.8	Maxillary sinus and mandibular ramus
Fibrosarcoma	22	31.88	48	11.8	Neck, maxillary sinus and mandibular ramus
UPS	8	11.59	42	6.1	Maxillary sinus and Nasal cavity
LGMS	2	2.9	52	9.5	Maxillary sinus and Lower neck
Carcinosarcoma	1	1.45	50	8	Upper alveolar bone
Chondrosarcoma	1	1.45	41	6	Sphenoid sinus
Neurofibrosarcoma	1	1.45	56	11	Lower neck
Hemangiosarcoma	1	1.45	42	5	Wing jaw
Undifferentiated sarcoma^c^	9	13.04			

*UPS* undifferentiated pleomorphic sarcoma, *LGMS* low-grade myofibroblastic sarcoma

^a^The median age at the diagnosis of radiation-induced sarcomas

^b^The most common site

^c^The results of aspiration biopsy, the only diagnosis of sarcoma, unable to perform further classification

**Table 4:** Subgroup comparison of radiological findings between osteosarcoma and fibrosarcoma

Histology	No.	Site^‡^	Extent		Margin definition		Bone destruction		Bone formation		Density/signal intensity un/enhanced tumor	
			Yes	No	Ill	Well	Yes	No	Yes	No	Heterogeneous	Homogeneous
Osteosarcoma	24	Maxillary sinus and mandibular ramus	20	4	21	3	23	1	10	14	24	
Fibrosarcoma	22	Neck, maxillary sinus and mandibular ramus	14	8	16	6	9	13			22	

^‡^The most common sites

### Treatment and clinical follow-up

Treatment and outcomes of the RIS are shown in Table 5. There were 60 patients receiving varied managements in our hospital. Apart from 8 patients having no follow-up data, a total of 46 patients had recurrent or uncontrolled lesions exactly at the original site of RIS until the end of the study/follow-up, accounting for 88.5 %. 47 patients received surgery, among them 5 patients received adjuvant chemotherapy or RT and 36 patients had recurrent lesions. The median latency from the surgery to the diagnosis of recurrence was 5.5 months (average 9.1 months, range 0.5–54 months). All of the other 10 patients who received chemotherapy only (*n* = 8), RT only (*n* = 1) or chemoradiation (*n* = 1) had uncontrolled lesions.

**Table 5:** Treatment and outcomes of radiation-induced sarcomas (*n* = 69)

Outcomes	Treatment						
	Surgery	Chemotherapy	Radiotherapy (RT)	Surgery + chemotherapy	Surgery + RT	Chemoradiation	No treatment
No. of cases	42	10	2	1 (preoperative chemotherapy) + 3 (postoperative chemotherapy)	1 (postoperative RT)	1	9
Recurrent/uncontrolled	31	8	1	4	1	1	
No recurrence	6						
Missing follow-up	5	2	1				
Latency of recurrence (months)	9.5 (0.5–54)			7.7 (1–18)	3		
Chemotherapy regimen		Varied		Varied			
Radiation dose (Gy)			70		70	56	

## Discussion

Patients with NPC treated with radiotherapy are at risk of developing radiation-induced sarcomas in the irradiated fields. RIS in NPC are even rare, with a crude incidence of 0.25 % in this study, which is generally consistent with previous findings. In this study, we retrospectively reviewed the clinical and CT/MRI features of RIS, and the results showed that RIS was very aggressive and had poor prognosis for most cases. Here, one thing to say is that among the 69 patients enrolled in this study, 54 had been documented focusing on the locations and histological subtypes of RIS in one of our previous studies [2].

We acknowledge that not all sarcomas that arise after RT are induced by radiation, so do the cases in our study; but it is impossible to differentiate RIS from primary sarcomas. We defined an RIS with a latency of at least 3 years after RT mentioned above. RIS in this study arose as a late complication of radiotherapy at an average latency period of 10.8 years, which was in agreement with the previously reported latency periods ranging from 8.8 years to 12.4 years [1–3, 13]. As many as 87.0 % of RIS developed within 15 years after RT, and the longest latency was up to 37 years after RT, which indicates that a long period of attention on RIS for patients treated with RT for NPC is necessary. But it should be noted that intensive follow-up is not necessary; patients just need to comply with the prescribed periodic follow-ups for NPC by radiation oncologist; and we radiologists should carefully scrutinize the scans, especially the irradiated field, to avoid missing any potential RIS lesions.

RIS had a predilection for arising in the high-radiation-dose region, and it is known that a total dose of 55 Gy or above increases their risk [1, 14]; our results supported the published findings. Patients undergoing a full course of RT for NPC received an average dose of about 68.7 Gy to the nasopharynx in this study, thus putting the surrounding bones and soft tissues at risk of RIS. The dose of radiation increases further in cases where there is local tumor invasion into the parapharyngeal region [15]. In this series, the most common origin site of RIS was the maxillary region, which encompasses at least the posterior half of the maxillary sinus and adjacent nasal cavity receiving almost the full radiation dose; and followed by the neck which received an average dose of about 57.7 Gy with lymph node0 involvement. We documented a similar observation that had been made previously [1–3, 9]. This highlighted the importance of inspecting the previously irradiated fields, especially the high-radiation-dose regions, on CT and (or) MRI follow-up scans to improve early detection of RIS.

Despite the small number of cases in this study, a wide variety of sites and subtypes of RIS were seen. Some of the patients from January 2000 to December 2011 had been reported by Cai et al. focusing on the imaging findings of RIS [2]. Patients in this study were collected from December 1998 to September 2012; additionally, we summarized the treatment and outcomes of RIS in our institution, some of the patients included in the previous study had been excluded in this study owing to missing clinical data or treated in other institutions. As a result, the most common locations and frequency number of histologic types of RIS showed some difference in this study. In addition to some osteosarcomas with tumor bone formation, the other sarcomas had almost similar appearances on CT or MRI. The hallmark of RIS was heterogeneous soft tissue mass with marked contrast enhancement. Most sarcomas in the series were large with extensive local invasion at the time of CT or MRI, 30.4 % of which were ≥5 cm. This is partly because they are very aggressive tumors that often grow rapidly.

The clinical diagnosis of RIS in the head and neck can be difficult because of induration and fibrosis within the irradiated field; this was also part of the reasons for the late diagnosis of most RIS. Prompt investigation based on the patient’s symptoms, especially the appearance of or a change in the character of, pain or a mass in the irradiated area several years after RT, could lead to an early diagnosis. Heterogeneous soft tissue masses with large size, extensive local invasion and (or) bony destruction in the irradiated fields on imaging examinations are the main clues to the diagnosis of RIS; if possible, histopathological results are needed.

Most RIS in the series were advanced at diagnosis, leading to a poor prognosis. Apart from eight patients having missing follow-up, 88.5 % of patients had recurrent or uncontrolled lesions exactly at the original site of RIS, which was consistent with data from other studies [13, 16–18]. It is generally accepted that complete surgical resection provides the only chance of cure [13, 16–19], but most sarcomas were not resectable because of their deep-seated location, close to vital structures, and advanced stage at the time of diagnosis. Imaging may potentially improve prognosis by allowing the detection of RIS at an earlier stage and by providing preoperative mapping of the complete extent of the sarcomas. Besides, sarcomas did not respond well to RT or chemotherapy [3, 4, 17]; RIS receiving RT or (and) chemotherapy only in this study showed worse results of persistent lesions.

A few limitations are identified in this study. Firstly, the estimated prevalence did not reflect the true number of RIS in post-RT NPC patients. This is because some patients undergo CT/MRI examinations and treatments in other institutions. That is to say, unfortunately, we had reported insufficient data to a certain extent. Secondly, it is acknowledged that some of the carcinomas may have been the second primary tumors rather than RIS, although our arguments for believing they are related to radiation have been discussed. Thirdly, patients in this study received conventional radiotherapy rather than IMRT. It is possible that IMRT with the delivery of a higher integral dose and also the additional effect of chemotherapy (neoadjuvant and/or concurrent chemotherapy) in some patients may also impact on the risk of developing RIS. Further studies are needed to explicitly state the issue.

## Conclusion

The current retrospective study confirmed the wide variety of locations and histological subtypes, latency as well as poor prognosis of RIS in the head and neck after RT for NPC. The imaging findings are not diagnosis specific, but all RISs were heterogeneous and well-enhanced mass. Radiologists should carefully scrutinize each scan, especially the irradiated field, to avoid missing any potential RIS lesions. For patients with NPC who received RT, when any clinical features suggest deterioration in the irradiated area, a prompt investigation, including an imaging examination when necessary, may help to provide an earlier diagnosis of RIS.

## Abbreviation

RT: Radiotherapy
NPC: Nasopharyngeal carcinoma
SCC: Squamous cell carcinoma
RIS: Radiation-induced sarcomas
CT: Computed tomography
MRI: Magnetic resonance imaging
